# The Urinary Level of Injury Biomarkers Is Not Univocally Reflective of the Extent of Toxic Renal Tubular Injury in Rats

**DOI:** 10.3390/ijms23073494

**Published:** 2022-03-23

**Authors:** Sandra M. Sancho-Martínez, María Herrero, Miguel Fontecha-Barriuso, Joana Mercado-Hernández, Francisco J. López-Hernández

**Affiliations:** 1 Institute of Biomedical Research of Salamanca (IBSAL), 37007 Salamanca, Spain; smsanchom@usal.es (S.M.S.-M.); joana.mercado.h@usal.es (J.M.-H.); 2 Departamento de Fisiología y Farmacología, Universidad de Salamanca (USAL), 37007 Salamanca, Spain; mariahg@usal.es (M.H.); fontechab@gmail.com (M.F.-B.); 3 Group of Translational Research on Renal and Cardiovascular Diseases (TRECARD), 37007 Salamanca, Spain; 4 National Network for Kidney Research REDINREN, RD016/0009/0025, Instituto de Salud Carlos III, 28029 Madrid, Spain; 5 Fundación Instituto de Estudios de Ciencias de la Salud de Castilla y León (IECSCYL), 42002 Soria, Spain; 6 Group of Biomedical Research on Critical Care (BioCritic), 47003 Valladolid, Spain

**Keywords:** acute kidney injury, urinary biomarkers, plasma creatinine, tubular injury, tubular necrosis

## Abstract

Nephrotoxicity is a major cause of intrinsic acute kidney injury (AKI). Because renal tissue damage may occur independently of a reduction in glomerular filtration rate and of elevations in plasma creatinine concentration, so-called injury biomarkers have been proposed to form part of diagnostic criteria as reflective of tubular damage independently of renal function status. We studied whether the urinary level of NGAL, KIM-1, GM2AP, t-gelsolin, and REGIIIb informed on the extent of tubular damage in rat models of nephrotoxicity, regardless of the etiology, moment of observation, and underlying pathophysiology. At a time of overt AKI, urinary biomarkers were measured by Western blot or ELISA, and tubular necrosis was scored from histological specimens stained with hematoxylin and eosin. Correlation and regression studies revealed that only weak relations existed between biomarkers and tubular damage. Due to high interindividual variability in the extent of damage for any given biomarker level, urinary injury biomarkers did not necessarily reflect the extent of the underlying tissue injury in individual rats. We contended, in this work, that further pathophysiological contextualization is necessary to understand the diagnostic significance of injury biomarkers before they can be used for renal tubular damage severity stratification in the context of nephrotoxic and, in general, intrinsic AKI.

## 1. Introduction

Acute kidney injury (AKI) is a syndrome characterized by abrupt decline in renal excretory function derived from reduction of the glomerular filtration rate (GFR), causing azotemia, alterations in diuresis (usually oliguria), or both, with important sanitary and economic consequences [1]. Accounting for 60–70% of cases, pre-renal AKI is the most frequent form, and is caused by altered renal hemodynamics and decreased net filtration pressure [2]. Purely pre-renal AKI courses with no damage to the renal tissues, but with azotemia and uremia that usually normalize after hydration and withdrawal of the cause with no apparent sequelae [3,4,5]. However, the most serious form is renal AKI, which develops as a consequence of primary renal parenchymal injury, caused by drugs, toxins, ischemia, etc., with longer and more complicated resolutions [3].

The typical and most common injury pattern of renal AKI is termed acute tubular injury (ATI) [6], comprising the older concept of acute tubular necrosis. ATI encompasses a wide range of functional and histological alterations, primarily affecting renal tubule epithelial cells. Tubular dysfunction and impaired tubular reabsorption may result from subtle (or not apparent) alterations in tubule epithelial cell morphology that do not compromise cell viability, such as cellular vacuolation, from loss of brush border or polarity, or from more or less extended epithelial cell death. Tubular cell death is often associated with tubular obstruction produced by luminal accumulation of tissue debris [7]. Impaired tubular reabsorption activates the tubuloglomerular feedback mechanism, which reduces glomerular filtration dramatically to prevent massive water and electrolyte loss [8]. Tubular obstruction also reduces filtration by partially or completely voiding the contribution of affected nephrons to overall filtration and favors tubulo-capillary backleak [7]. Following cell injury and death, activated tubular and interstitial cells rampage on production of vasoactive mediators [9,10,11], which, in turn, cause mesangial cell contraction and vasoconstriction contributing to lowering GFR and renal excretory function.

International criteria for AKI definition and diagnosis (e.g., RIFLE, AKIN, KDIGO) rely on increments in plasma creatinine concentration (Cr_p_) or reductions in urinary output [1]. Cr_p_ is a poor and late surrogate of GFR and is practically useless for AKI diagnosis in clinical circumstances until over 60% of renal excretory function is lost [12]. Cr_p_ is also highly unspecific at informing on the underlying pathophysiological mechanisms, and the extent or absence of tissue injury. For instance, Cr_p_ may increase with (as in ATI) or without (as in pre-renal AKI) underlying tissue damage. Similarly, extensive renal damage may occur without concomitant elevations in Cr_p_ [13]. Because of these limitations, a number of injury markers have been erupting into the clinical scenario. They have been identified for their capacity to detect AKI before—and independently of—renal function status and plasma creatinine level [14,15,16,17]. These new biomarkers include, most notably, kidney injury molecule (KIM-1) and neutrophil gelatinase-associated lipocalin (NGAL), tissue inhibitor of metalloproteinase 2 (TIMP-2) and insulin-like growth factor binding protein 7 (IGFBP7) [13,18,19]. Their diagnostic utility has been proposed to derive from their increased expression in the damaged renal tissue and their ready transfer to bodily fluids (i.e., the blood and urine), long before ATI is extensive enough to make Cr_p_ increase [20,21,22,23,24]. This has led to their being termed injury biomarkers, indicative of renal tissue damage, as differentiated from renal function (functional) biomarkers such as GFR and Cr_p_ [25]. We have also identified new urinary biomarkers with additional diagnostic characteristics for AKI diagnosis, including ganglioside M2 activator protein (GM2AP) [26,27], t-gelsolin (i.e., a cleavage product of gelsolin produced by apoptotic caspases) [28,29] and regenerating islet-derived IIIb (REGIIIb) [28], which associate with renal forms of AKI.

However, it remains uncertain whether the urinary level of these biomarkers reflects tissue damage, functional damage, or a combination of both, and whether biomarker level is indicative of the extent of injury. Early-stage diagnosis of damage and of the extent of injury is critical for timely intervention (i.e., cause withdrawal and supportive treatment), and patient prognosis [30,31,32]. With this background in mind, we analyzed the relationship between the level of different urinary biomarkers and the extent of renal tissue damage and renal dysfunction during AKI in rats, irrespective of other factors such as etiology and disease stage. Our approach aimed to model the interpretation of clinical AKI cases of undetermined cause and status at the time of potential biomarker-based diagnosis.

## 2. Results

### 2.1. The Study Population Provides a Spectrum of ATI Patterns and Damage Severity

**Figure 1:**
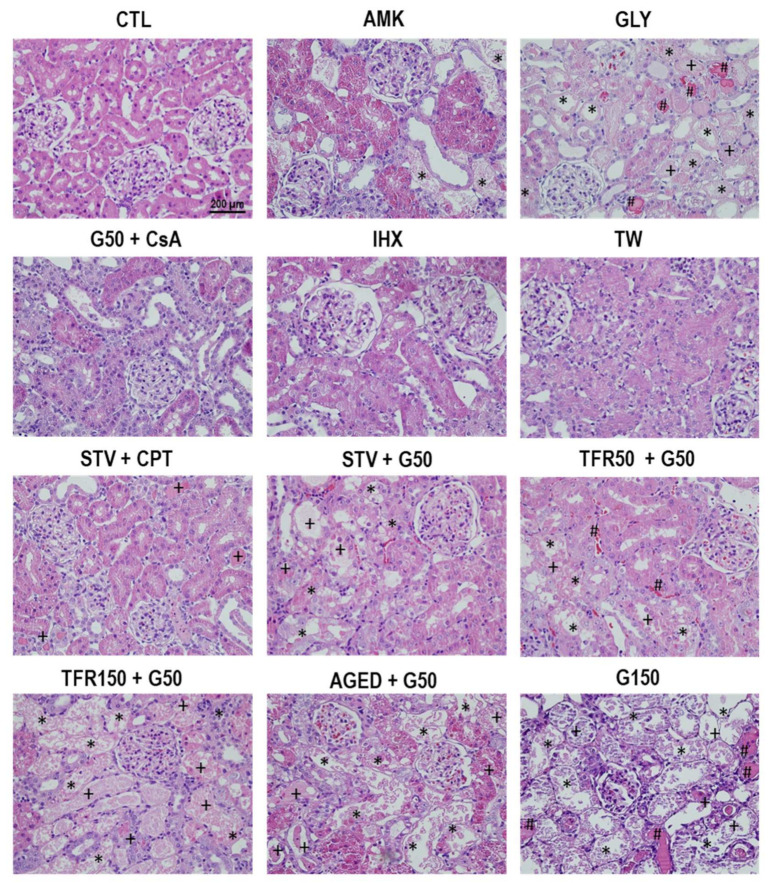
Cortical histology. Representative images of the renal cortex of rats from the AKI models included in the study. (+ luminal debris, * tubular necrosis, # tubular obstruction).

**Figure 2:**
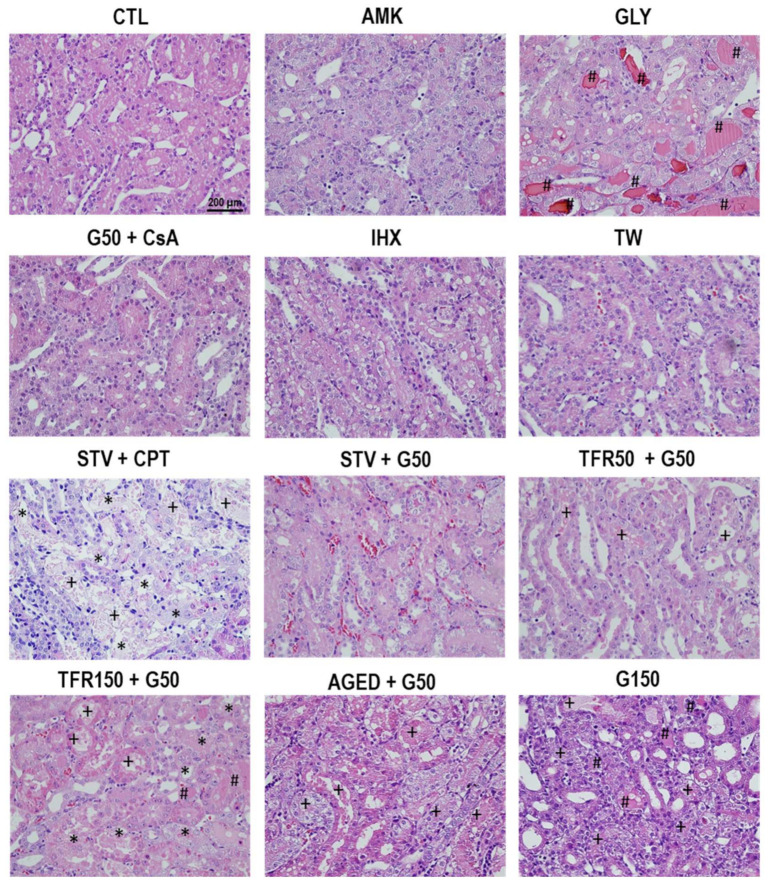
Outer medullary histology. Representative images of the renal outer medulla of rats from the AKI models included in the study. (+ luminal debris, * tubular necrosis, # tubular obstruction).

The renal insults used to induce AKI in the animals of this study generated different degrees of acute tubular injury affecting mostly cortical or outer medullary nephron segments, depending on the drug or drug combination. Figure 1 and Figure 2 show representative images of the cortical and outer medullary regions, respectively, of the kidneys from each model. Deeper into the medulla, no significant damage occurred in any of the models. Only accumulation of intratubular hyaline material (likely composed of debris from upstream epithelial derangement) was observed in the internal medulla. Table 1 shows a summary of the typical histological findings of each experimental model. A variety of damage patterns and severity was observed among AKI models, ranging from no parenchymal injury to severe tubular necrosis. This diversity provided an ample substratum on which to study the relationship between the urinary level of different AKI biomarkers and the extent of tubular damage, independently of AKI etiopathology or AKI stage.

### 2.2. The Urinary Level of Injury Biomarkers Weakly Associates with Tubular Damage Regardless of AKI Etiopathology or Stage

**Figure 3:**
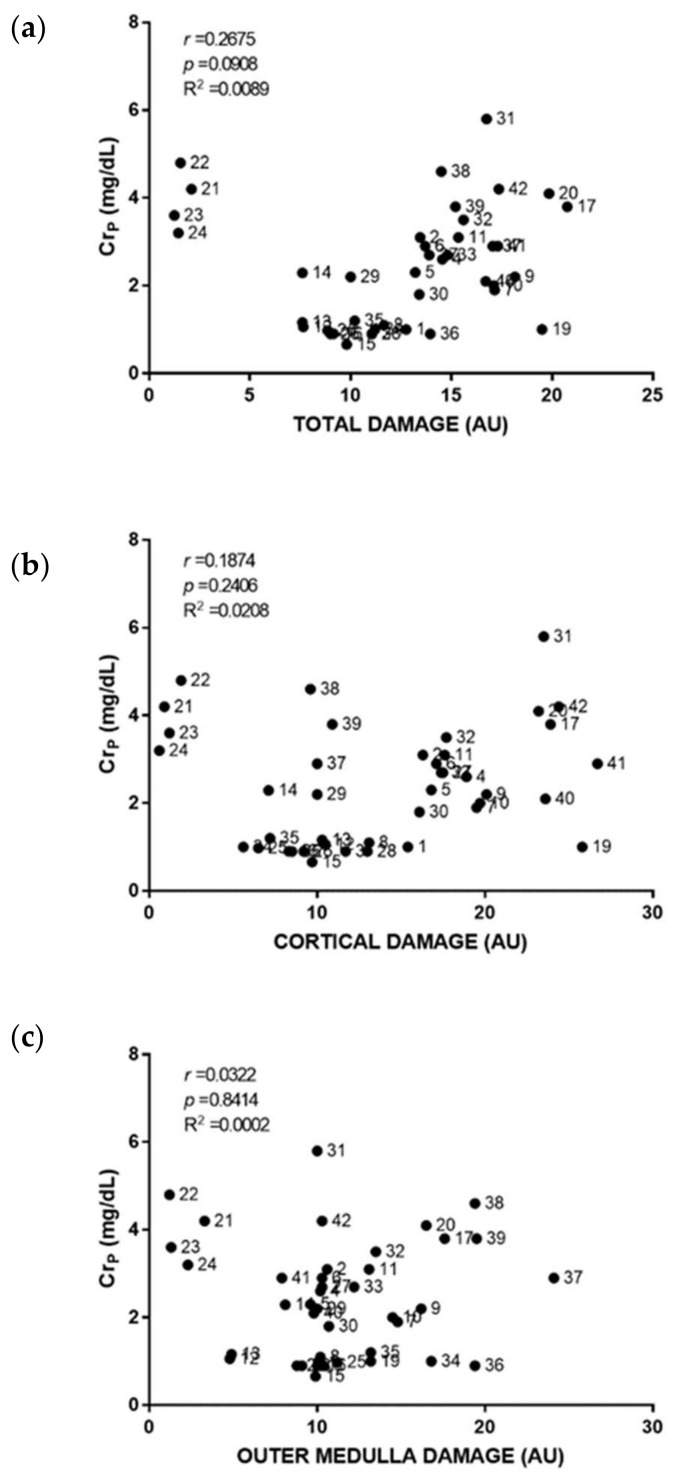
Plasma creatinine to renal damage relationship. Scatter plots showing the relationship between plasma creatinine concentration (Cr_p_) with overall renal tissue damage (**a**), renal cortical damage (**b**), and outer medullary damage (**c**) of individual rats. AU: arbitrary units. *p*: Spearman’s correlation *p*-value. *r*: Spearman’s correlation coefficient. R^2^: linear regression coefficient of determination.

**Figure 4:**
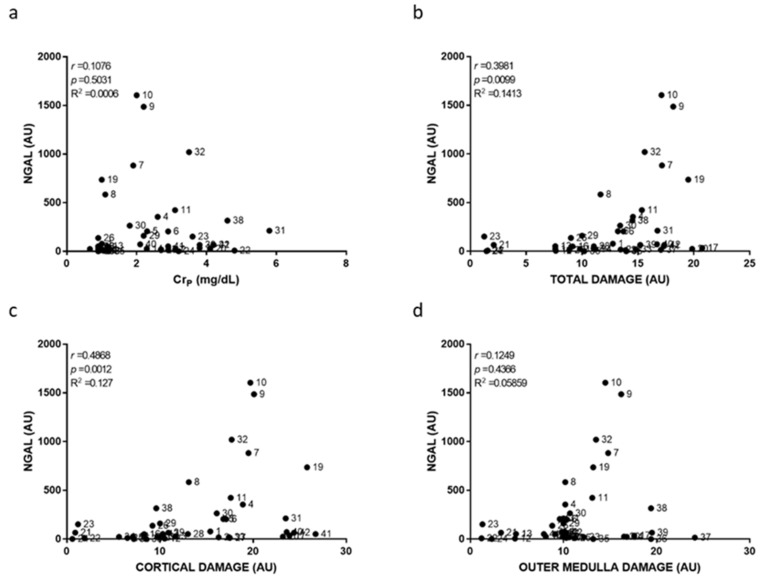
Urinary NGAL to plasma creatinine and to renal damage relationships. Scatter plots showing the relationship between urinary NGAL levels and plasma creatinine concentration (Cr_p_) (**a**), overall renal tissue damage (**b**), renal cortical damage (**c**), and outer medullary damage (**d**) of individual rats. AU, arbitrary units. *p*, Spearman’s correlation *p*-value. *r*, Spearman’s correlation coefficient. R^2^, linear regression coefficient of determination.

**Figure 5:**
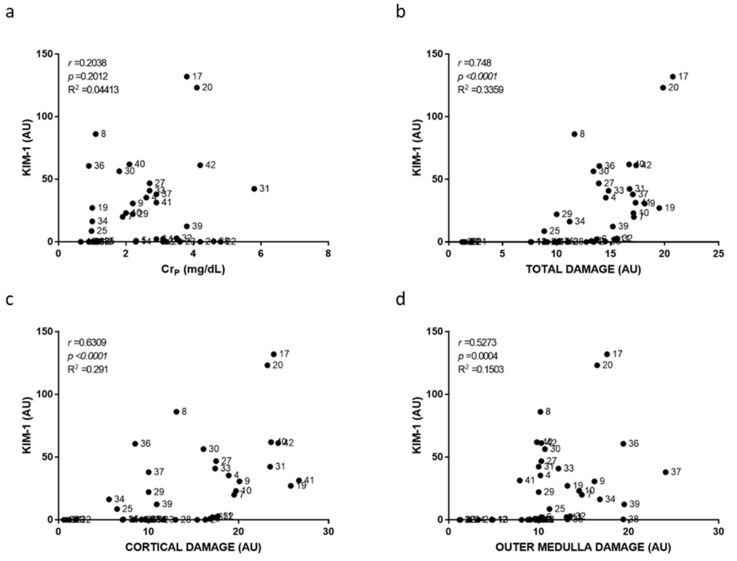
Urinary KIM-1 to plasma creatinine and to renal damage relationships. Scatter plots showing the relationship of urinary KIM-1 level with plasma creatinine concentration (Cr_p_) (**a**), with the overall renal tissue damage (**b**), with renal cortical damage (**c**), and with outer medullary damage (**d**) of individual rats. AU, arbitrary units. *p*: Spearman’s correlation *p*-value. *r*: Spearman’s correlation coefficient. R^2^: linear regression coefficient of determination.

**Figure 6:**
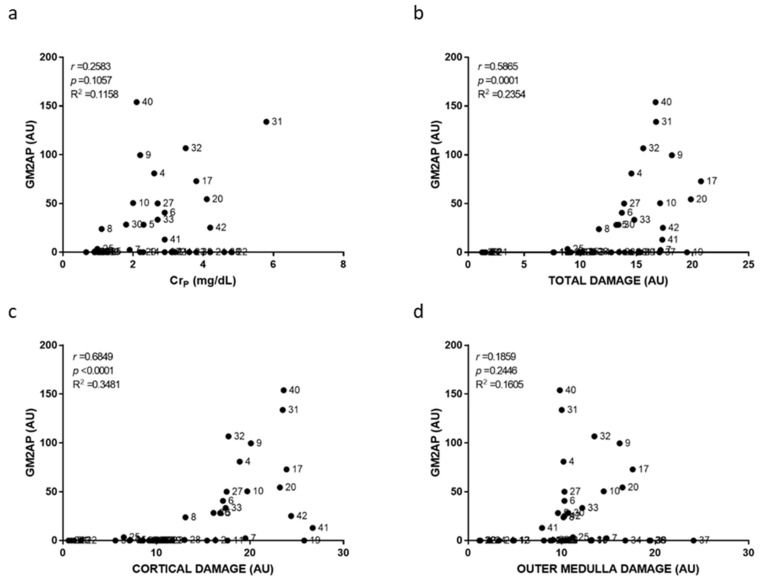
Urinary GM2AP to plasma creatinine and to renal damage relationships. Scatter plots showing the relationship between urinary GM2AP levels and plasma creatinine concentration (Cr_p_) (**a**), overall renal tissue damage (**b**), cortical damage (**c**), and outer medullary damage (**d**) of individual rats. AU: arbitrary units. *p*: Spearman’s correlation *p*-value. *r*: Spearman’s correlation coefficient. R^2^: linear regression coefficient of determination.

**Figure 7:**
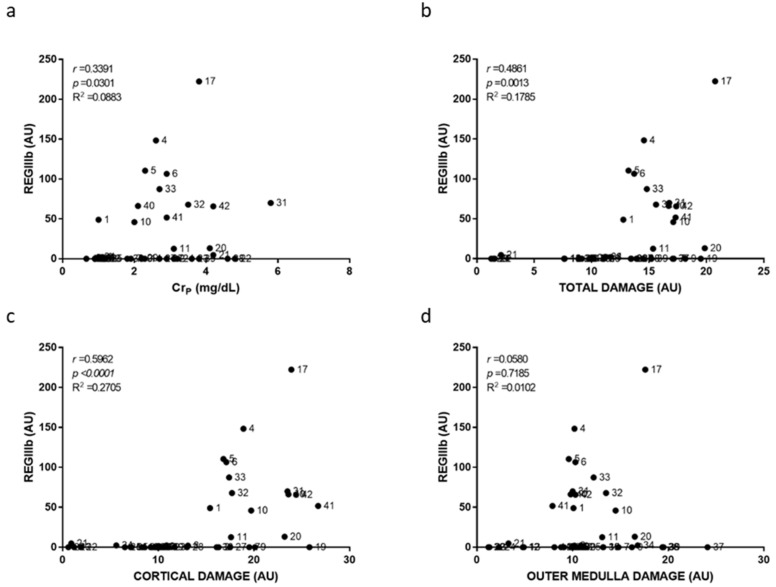
Urinary REGIIIb to plasma creatinine and to renal damage relationships. Scatter plots showing the relationship between urinary REGIIIb levels and plasma creatinine concentration (Cr_p_) (**a**), with the overall renal tissue damage (**b**), renal cortical damage (**c**), and outer medullary damage (**d**) of individual rats. AU: arbitrary units. *p*: Spearman’s correlation *p*-value. *r*: Spearman’s correlation coefficient. R^2^: linear regression coefficient of determination.

**Figure 8:**
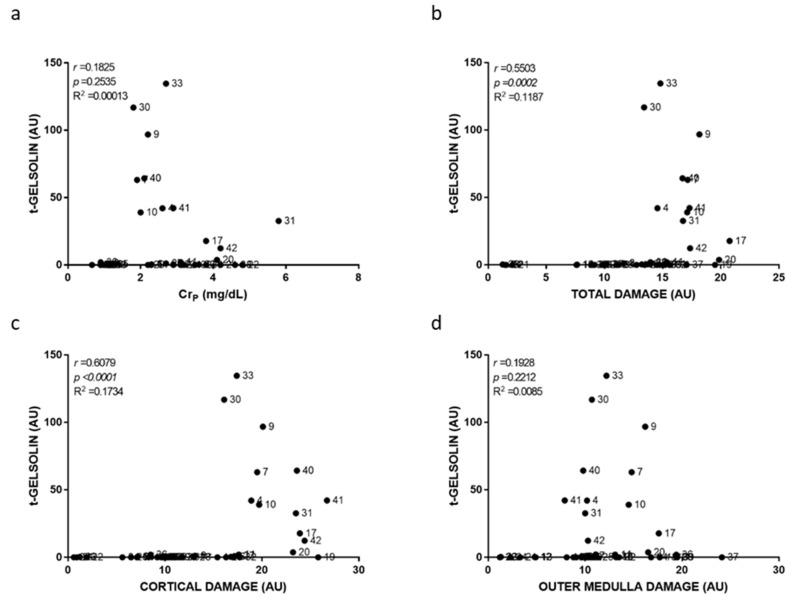
Urinary t-gelsolin to plasma creatinine and to renal damage relationships. Scatter plots showing the relationship between urinary t-gelsolin levels and plasma creatinine concentration (Cr_p_) (**a**), overall renal tissue damage (**b**), renal cortical damage (**c**), and outer medullary damage (**d**) of individual rats. AU: arbitrary units. *p*: Spearman’s correlation *p*-value. *r*: Spearman’s correlation coefficient. R^2^: linear regression coefficient of determination.

Individual damage scoring was used to plot and relate cortical, outer medullary and total damage to individual biomarker level in Figure 3, Figure 4, Figure 5, Figure 6, Figure 7 and Figure 8. As a general result, we found statistically significant (i.e., very low *p*-values) but weak (i.e., low r coefficients) Spearman correlations of some biomarkers with cortical and total damage. Linear regression studies provided very low fitting (i.e., very low R-squared coefficients) in all cases. This indicated that, although some tendencies might exist between the levels of some of these biomarkers and the extent of renal cortical damage, the high variability weakend their potential use as renal damage intensity probes. As expected from the definition of AKI itself, Cr_p_ showed a very weak and non-significant relation with renal damage (Figure 3). All the urinary biomarkers studied showed weak but statistically significant correlations with both cortical and total renal damage. The cortex was the most injured area and thus had the greatest overall damage in most models, which could explain the parallelism of cortical and overall damage correlations. KIM-1 was the only biomarker studied whose urinary levels showed significant correlation with medullary damage; however, linear regression fitting was very low. Finally, all urinary biomarkers studied had their lowest, statistically non-significant correlations and insignificant linear regression fitting with Cr_p_.

## 3. Discussion

**Figure 9:**
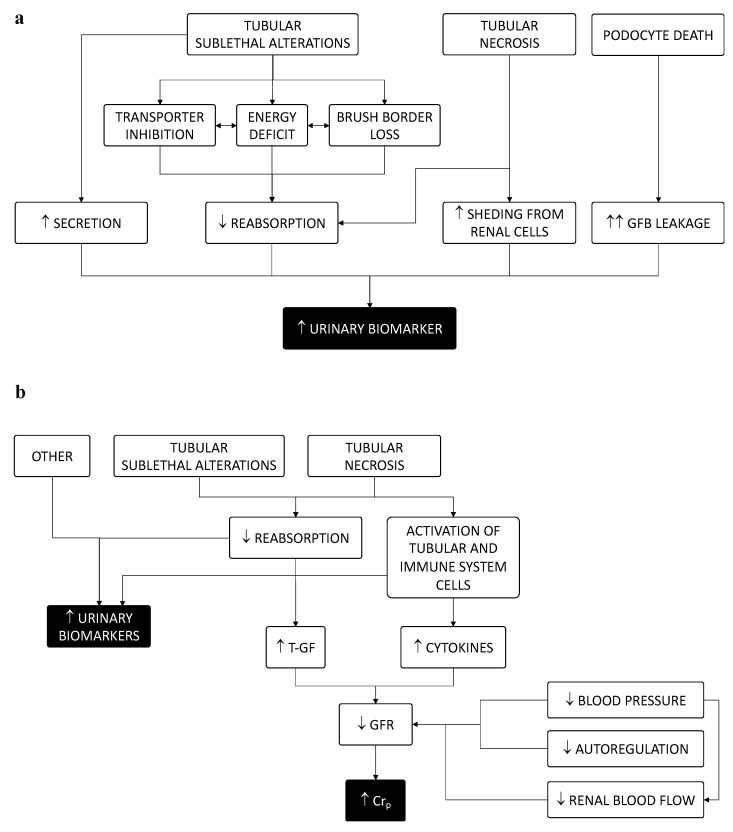
(**a**) Mechanisms potentially involved in increased biomarker urinary excretion. (**b**) Interplay between mechanisms causing reductions in GFR and those leading to increased biomarker excretion. Cr_p_: plasma creatinine concentration. GFB: glomerular filtration barrier. GFR: glomerular filtration rate. T-GF: tubule-glomerular feedback. ↑: increase. ↓: decrease.

The results of this study showed that only weak group associations were found between the urinary level of the so-called injury biomarkers NGAL and KIM-1. This was also true of ATI-associated biomarkers GM2AP, t-gelsolin and REGIIIb, and the degree of renal tubular injury in rats. The high interindividual variability indicated that urinary biomarker levels did not univocally inform on the extent of tissue damage at the individual level. This implied that, beyond tissue damage, additional factors derived from individual or etiopathological differences contributed to the renal excretion of these biomarkers. In fact, biomarkers may be excreted with the urine through different mechanisms, all of which could be altered under pathological conditions. Sources of elevated urinary biomarkers included: increased shedding from renal cells (or from the lower urinary tract), increased filtration from the bloodstream (overwhelming the tubular reuptake capacity), higher tubular secretion, decreased tubular reabsorption, and combinations thereof (Figure 9a). Increased filtration could occur following disruption of the glomerular filtration barrier leading to leakage of proteins normally bound into the bloodstream, rather than following increased GFR, which drops during AKI. Further granularity may also contribute to explaining decoupling from damage and biomarkers as well as differences among biomarkers. In fact, alterations in shedding, reabsorption or secretion may involve different renal compartments or tubule segments from biomarker to biomarker or, as in the case of NGAL, may be produced by a segment (i.e., distal tubules) following injury in another (i.e., proximal) area [33].

In a classical view, renal tissue injury is thought to contribute to increasing shedding of injury-related biomarkers from renal (mostly tubular) cells and to reduce reabsorption (or both). However, biomarker shedding and handicapped reabsorption, leading to increased biomarker urinary excretion, may also result, for instance, from sublethal alterations not distorting renal tissue architecture, i.e., not causing tissue injury. This situation is exemplified by several models wherein defective reabsorption occurs without concomitant tissue injury. These include the maleate model and the megalin knock-out model. Acute administration of sodium maleate uncouples proximal transmembrane transport and inhibits proximal reabsorption [34,35,36], leading to increased urinary excretion of a variety of protein biomarkers in the ultrafiltrate, with no effect on renal structures [26,34,37,38,39,40]. A very similar scenario is generated by knocking out megalin [41], an essential member of the proximal multiligand binding receptor endocytic complex responsible for protein reclamation [42]. Indeed, iconic biomarkers such as NGAL [43,44], TIMP-2 and IGFBP7 [45], long believed to be shed directly by damaged tubules and thus considered injury biomarkers, have been shown to appear in the urine as a consequence of handicapped reabsorption [38,46,47]. Their diagnostic application has both gained granularity and changed its pathophysiological significance. Sublethal alterations would thus explain elevations in urinary biomarkers without structural damage and void the implicit interpretation of increased injury biomarker as a sign of tissue derangement.

On the contrary, cases of extensive tissue injury with low (or normal) urinary biomarker levels remain unexplained. Hypothetically, very damaged nephrons may become obliterated by luminal tissue debris accumulation. Obliterated nephrons would not contribute to the overall urine and would thus not communicate pathological status information through urinary biomarkers. However, this did not seem to be the case in our models, because rats showing severe renal tissue injury and low urinary biomarker levels varied from one biomarker to another. This observation pointed at a distinct behavior of each biomarker as a function of tissue injury, which needs to be further investigated.

The level of urinary biomarkers was not linked to Cr_p_, and thus was not directly proportional to the filtration function (i.e., of GFR) (Figure 9b). In turn, no straightforward relationship existed between Cr_p_ and tissue damage, as observed in the introduction of this paper. Altogether, we do not contend that there is no relationship between tubular injury, GFR (and Cr_p_) and urinary biomarkers, but merely that this relationship is not directly proportional. In fact, damage was related to both urinary biomarkers and GFR (and Cr_p_), but additional factors decoupled these relationships. Thus, more biomarkers are not synonymous with more tubular damage, nor vice versa (Figure 9b). This dissociation has significant diagnostic and prognostic implications, as it weakens the relations between biomarkers, clinical phenotypes (of AKI) and clinical outcomes, at least in nephrotoxic models of AKI. Other models reproducing different acute and chronic etiopathologies and injury patterns must also be studied to gain broader insight into the applicability of the present results.

## 4. Materials and Methods

Unless otherwise indicated, all reagents were purchased from Sigma-Aldrich (Madrid, Spain).

### 4.1. Animals and Experimental Protocol

We used plasma, urine and renal tissue samples from past experiments performed in our laboratory involving a variety of toxic AKI models of ATI. The animals providing samples to this study are described in Table 2. They were not selected to represent specific models or categories, but to provide a heterogeneous casuistic collection with regard to pathophysiological patterns of tubular injury and degrees of damage, irrespective of etiology. In all cases, samples were obtained from male Wistar rats (220–240 g) housed under controlled experimental conditions and allowed free access to regular chow and water. Rats were treated in accordance with the Declaration of Helsinki and Principles on the Advice on Care and Use of Animals referred to in the 2010/63/UE law, and in the current Spanish legislation for experimental animal use and care, i.e., the Royal Decree 53/2013 of 1 February, published in the Official Bulletin of the State 34, Sec. I, page 11370–11421, 8 February 2013). All procedures were approved by the Bioethics Committee of the University of Salamanca and the Ministry of Agriculture and Livestock of the Regional Government of Castile and Leon (Spain), code 251 (approved on 18 June 2018), and code 494 (approved on 01 October 2020). Rats were allocated in metabolic cages to obtain 24 h individual urine samples. Blood was drawn with a needle from the tail vein, and plasma was obtained by centrifugation. Both urine and plasma samples were kept at −80 °C for later biochemical determinations. Immediately before sacrifice (which occurred by exsanguination under anesthesia at the end of the specified treatment periods, Table 2), rats were anesthetized with 50 mg/kg sodium pentobarbital, and the kidneys were in situ perfused with saline, dissected, and fixed in 3.7% *p*-formaldehyde for histological studies. In all cases, injury biomarkers were measured by Western blot or ELISA (as described below, Section 4.4 and Section 4.5) in urine samples taken the day of sacrifice and were thus contemporary with the histological analysis (see below, Section 4.2).

### 4.2. Renal Tissue Histopathology

Paraffin blocks were made with fixed kidneys, and 5-μm tissue sections were stained with hematoxylin and eosin for visual inspection. Photographs were taken under an Olympus BX51 microscope connected to an Olympus DP70 color, digital camera (Olympus, Tokyo, Japan). Tubular injury was blindly determined by means of an injury score, which was calculated semi-quantitatively, as previously described [37]. In total, 10 cortical and 10 outer medullary fields from each specimen were photographed, and each field was divided into 10 identical sections. A score of 0–3 was assigned to each section, according to the following criteria: 0, normal histology; 1, up to one third of the section affected by damage; 2, same as for 1, but from one third to two thirds of the section affected; and 3, the whole field affected. Section scores were added to give a field score (maximal score per field = 30). The average score of the 10 fields was assigned to each specimen. Damage was considered when findings of tubular necrosis and cell sloughing, tubular dilation, or presence of hyaline deposits were observed.

### 4.3. Plasma Creatinine Measurement

Plasma creatinine concentration was determined with a commercial colorimetric kit (Quantichrom Creatinine Assay Kits, BioAssay Systems, Hayward, CA, USA), according to the manufacturer’s instructions.

### 4.4. Western Blot

A volume of urine (21 μL) from each animal corresponding to the same fraction of their 24-h urinary output was separated by acrylamide electrophoresis. Proteins were transferred to an Immobilon-P Transfer Membrane (Merck-Millipore, Darmstadt, Germany) and incubated with antibodies against KIM-1 (RD systems, Minneapolis, MN, USA), t-gelsolin (Santa Cruz Biotechnology, Dallas, TX, USA), RegIIIB (RD systems, Minneapolis, MN, USA), GM2AP (a polyclonal antibody produced for our laboratory by Immunostep, Salamanca, Spain, as previously described (26)), followed by horseradish peroxidase-conjugated secondary antibodies and chemiluminiscent detection (Immobilon Western Chemiluminescent HRP Substrate kit, Millipore, Darmstadt, Germany) with photographic films (Kodak, Madrid, Spain). Bands were quantified by densitometric analysis with the Scion Image software (Frederick, MD, USA). A positive control was used in all experiments to normalize biomarker levels, and all biomarkers were expressed as arbitrary units (% of positive control, which was assigned the 100% value). Bands were quantified and normalized to the intensity of the positive control. The positive control was urine from a highly proteinuric rat urine which tested positive for the studied biomarkers in pilot studies.

### 4.5. ELISA

A commercial ELISA was used to measure NGAL (#KIT046, BioPorto Diagnostics, Hellerup, Denmark) according to the manufacturer’s instructions.

### 4.6. Statistical Analysis

Data were expressed as mean ± SEM. Linear regression analysis was used to study the relation of biomarker levels to the extent of renal damage or plasma creatinine concentration. The Kolmogorov–Smirnov test and visual inspection of histograms were used to evaluate data distribution (*p* values < 0.05 were considered non-normal). Because data displayed non-normal distributions, the Spearman’s correlation analysis was performed to evaluate the strength of relationship between two quantitative variables. GraphPad Prism 7.0 (GraphPad Software, San Diego, CA, USA) and IBM SPSS Statistics 25 software (IBM, Armonk, NY, USA) were used for the statistical analysis.

## 5. Conclusions

The level of urinary injury biomarkers did not necessarily reflect the extent of the underlying tubular injury, at least in rat models of nephrotoxic ATI. In humans, this relationship may be even more complicated due to a more pronounced interindividual phenotypic heterogeneity (compared to animal models) and biomarker pleiotropy associated with comorbidities potentially present in patients [48], which were absent in animal models. This study’s results served as a caution against interpretation of the diagnostic meaning of these biomarkers for kidney tubular damage stratification. Further study on the origin and traffic of individual biomarkers will be necessary to understand their true pathophysiological meaning and clinical application. Prospectively, precise damage contextualization and pathophysiological diagnosis of AKI and of AKI severity should involve the multidimensional, integrated analysis of biomarker panels in liquid (i.e., urinary) biopsies.

## Figures and Tables

**Table 1:** Renal histology of the AKI models. Typical renal cortical and medullary findings of the AKI models included in the study. LD: luminal debris. TN: tubular necrosis. TO: tubular obstruction. −: Normal histology (i.e., no pathological findings). Severity scale: +: low. ++: medium. +++: high. ++++: very high.

MODEL	CORTICAL			MEDULLARY		
	TN	LD	TO	TN	LD	TO
Control (CTL)	−	−	−	−	−	−
Amikacin (AMK)	+	+	−	−	−	−
Glycerol (GLY)	+++	++	++	−	−	++++
Gentamicin + Cyclosporin A (G50 + CsA)	−	+	−	−	−	−
Iohexol (IHX)	−	−	−	−	−	−
Tripple whammy (TW)	−	−	−	−	−	−
Starvation + Cisplatin (STV + CPT)	−	+	−	++++	+++	−
Starvation + Gentamicin (STV + G50)	+	++	−	−	−	−
Tenofovir + Gentamicin (TFR50 + G50)	++	+	−	−	+	−
Tenofovir+ Gentamicin (TFR150 + G50)	++++	++++	−	+	+++	++
Gentamicin aged rats (AGED + G50)	++++	++++	+	−	+	−
Gentamicin (G150)	++++	++++	++	−	++	++

**Table 2:** Animal characteristics. Animal model (i.e., AKI model), drug dose, and plasma creatinine concentration (Cr_p_) for each individual rat included in the study.

Group		Cr_P_ (mg/dL)	Treatment
No.	Acronym		
1	AMK	1.0	Amikacin (400 mg/kg/day) i.p.during 9 days
2	AMK	3.1	
3	AMK	0.9	
4	AMK	2.6	Amikacin (500 mg/kg/day) i.p.during 9 days
5	AMK	2.3	
6	AMK	2.9	
7	GLY	1.9	Glycerol (10 mg/kg) i.m. single dose
8	GLY	1.1	
9	GLY	2.2	
10	GLY	2.0	
11	GLY	3.1	
12	G50 + CsA	1.06	Cyclosporine (15 mg/kg/day) s.c. during 13 days and gentamicin (50 mg/kg/day) i.p .during the last 6 days
13	G50 + CsA	1.16	
14	IHX	2.29	Iohexol (3g/kg/day) i.v. a total of 3 doses, administered every other day
15	IHX	0.66	
16	IHX	0.91	
17	AGED + G50	3.8	Gentamicin (50 mg/kg/day) i.p.during 6 days, (9 month old rats)
18	AGED + G50	12.4	
19	AGED + G50	1.0	
20	AGED + G30	4.1	Gentamicin (30 mg/kg/day) i.p.during 6 days, (9 month old rats)
21	TW	4.2	Ibuprofen (400 mg/kg/day) plus trandolapril (0,7 mg/kg/day) v.o. during 10 days and furosemide (20mg/kg/day) i.p. during the last 6 days
22	TW	4.8	
23	TW	3.6	
24	TW	3.2	
25	STV + G50	0.98	Fasting for two days and followed by gentamicin (50 mg/kg/day) i.p. during 6 days
26	STV + G50	0.9	
27	STV + G50	2.7	
28	TFR50 + G50	0.9	Tenofovir (50 mg/kg/day) v.o. during 30 days and gentamicin (50 mg/kg/day) i.p .during the last 6 days
29	TFR50 + G50	2.2	
30	TFR50 + G50	1.8	
31	TFR150 + G50	5.8	Tenofovir (150 mg/kg/day) v.o. during 30 days and gentamicin (50 mg/kg/day) i.p .during the last 6 days
32	TFR150 + G50	3.5	
33	TFR150 + G50	2.7	
34	STV + CPT	1.0	Fasting for two days and then a single administration of cisplatin (2,5 mg/kg) i.p
35	STV + CPT	1.2	
36	STV + CPT	0.9	
37	STV + CPT	2.9	
38	STV + CPT	4.6	
39	STV + CPT	3.8	
40	G150	2.1	Gentamicin (150 mg/kg/day) i.p.during 6 days
41	G150	2.9	
42	G150	4.2	
43	CTL	0.5	Control (saline solution)
44	CTL	0.5	
45	CTL	0.6	

## Data Availability

Data are available to researchers upon reasonable request.
